# Proteomic genotyping of SNP of Complement Factor H (*CFH)* Y402H and I62V using multiple reaction monitoring (MRM) assays

**DOI:** 10.1038/s41598-022-20936-8

**Published:** 2022-11-15

**Authors:** Kyoung Lae Kim, Hyerim Kim, Youngju Lee, Cheolju Lee, Kwangsic Joo, Sang Jun Park, Kyu Hyung Park, Seong-Jun Park, Se Joon Woo

**Affiliations:** 1 Department of Ophthalmology, Seoul National University College of Medicine, Seoul National University Bundang Hospital, 82, Gumi-ro 173 Beon-gil, Bundang-gu, Seongnam-si, Gyeonggi-do 13620 Republic of Korea; 2 Department of Ophthalmology, Kangdong Sacred Heart Hospital, Hallym University College of Medicine, Seoul, Republic of Korea; 3 Department of Research, RetiMark Co., Ltd, 17, Seobinggo-ro, Youngsan-gu, Seoul, 04387 Republic of Korea; 4 Center for Theragnosis, Korea Institute of Science and Technology, Seongbuk-gu, Seoul, Republic of Korea; 5 Department of Ophthalmology, Seoul National University Hospital, Seoul, Korea

**Keywords:** Molecular biology, Medical research, Diagnostic markers

## Abstract

The single nucleotide polymorphisms (SNPs) of complement factor H (CFH) gene are well-known genetic risk factors for age-related macular degeneration (AMD). To identify whether the measurement of plasma protein concentrations of *CFH* variants using the multiple reaction monitoring (MRM) assay can determine the genotypes of *CFH* SNP *rs*1061170 and *rs*800292, 120 patients with AMD and 26 controls were included in this study. The number of cases were TT:TC:CC = 121:24:1 in *CFH* SNP Y402H and GG:AG:AA = 72:57:17 in *CFH* SNP I62V. Plasma concentrations of tryptic peptides were measured using the MRM assay, and tyrosine/histidine (Y/H) and valine/isoleucine (V/I) CFH variant protein ratios were obtained. To discriminate the genotypes by the plasma protein ratios, cut-off values were set for Y/H ratios (TT: > 4.428; TC: 1.00–4.428; CC: < 1.00) and V/I ratios (GG: > 1.09; AG: 0.0089–1.08; AA: < 0.0089). Correlation analysis revealed that the plasma CFH variant protein ratios and genotypes of *CFH* were exactly matched (100%) without overlap in the total patients and controls. The measurement of plasma protein CFH variants using the MRM assay can accurately identify the genotypes of *CFH* SNPs of Y402H and I62V.

## Introduction

Age-related macular degeneration (AMD) is one of the major causes of blindness among adults aged 65 years or above in developed countries. Ageing, genetic factors, and smoking are well-known risk factors for AMD^1^.

AMD is a multifactorial disease and Inflammation is known to be one of the major mechanisms for the development and progression of AMD^2^. The complement system plays an important role in the mediation of inflammatory response and is considered to be related to the pathophysiology of AMD^3^. Previous genome-wide association studies and validation studies revealed that the variants of complement factor H (CFH) are strongly associated with the risk of AMD: risk allele of *CFH* single nucleotide polymorphism (SNP) *rs*1061170 (risk allele: C, tyrosine 402 to histidine [Y402H]) and *rs*800292 (risk allele: G, isoleucine 62 to valine [I62V])^4–9^.

To determine the genotypes of *CFH* SNPs, deoxyriboNucleic Acid (DNA) purification and sequencing are required. If the genotype could be revealed by blood protein quantification, it can be a useful tool and can be integrated with other protein markers while alleviating the need for DNA analysis. However, quantifying highly homologous sequence variants of *CFH* using the currently available antibody and aptamer-based approaches is not feasible. Multiple reaction monitoring (MRM) assay has emerged as a useful method for precise and accurate quantification of proteins and their variants that cannot be detected using antibody-based methods^10^. Recently, the detection of multiple *CFH* protein variants using MRM assays has been reported, showing the possibility that the *CFH* genotypes can be identified using MRM assay^11^.

In this study, to determine the actual clinical utility of MRM assay for *CFH* SNP genotyping, we analyzed the correlation between the genotype of the major *CFH* SNPs *rs*1061170 (Y402H) and *rs*800292 (I62V) and the concentration ratios of plasma *CFH* variants measured using the MRM assay in a cohort of AMD patients and normal controls. Additionally, we also evaluated the possibility of replacing genetic testing with the MRM assay for the risk assessment of AMD via proteomic *CFH* genotyping.

## Methods

This study protocol was reviewed and approved by the Institutional Review Board (IRB) of Seoul National University Bundang Hospital (IRB No: B-2105-682-105). In this study, a retrospective analysis was conducted on the collected clinical data and blood samples from the previous Bundang AMD Genetic Study^9^. The current study was conducted in accordance with the Declaration of Helsinki. A written informed consent was obtained from all participants in the secondary study.

### Participants

Participants aged over 50 years who were newly diagnosed with intermediate AMD and nAMD between October 1, 2003, and December 31, 2016, were enrolled in the AMD group in our study. Participants aged over 50 years who were newly diagnosed with cataracts, without any retinal pathology between October 1, 2003, and December 31, 2016, were enrolled as the control group in our study. Intermediate AMD was defined as the presence of multiple large drusen (> 125 µm) on the macula with or without pigmentary changes, the absence of pre-existing central geographic atrophy (GA), and nAMD based on the international AMD classification^12^. nAMD was defined as the evidence of choroidal neovascularization (CNV) associated with non-drusenoid retinal pigment epithelium detachment, serous sensory retinal detachment, subretinal hemorrhage, and/or subretinal exudation. We collected basic demographic information, including age and sex, at the first visit. Smoking status was classified as non-smoker or former/current smoker.

### DNA-based genotyping of *CFH* SNPs

Patient DNA was obtained from peripheral blood using a DNA extraction kit (QIAamp DNA Maxi kit, Qiagen Inc.). Multiplex polymerase chain reaction using a single base extension technique was performed for SNP genotyping using the iPLEX Gold kit and MassARRAY Typer software version 4.0 (Sequenom, San Diego, CA). Two SNPs known to be major risk alleles for AMD in Koreans, namely *CFH rs*1061170 and *rs*800292, were analyzed^9^.

### Selection of target tryptic peptides

For proteomic genotyping, it is necessary to determine how the SNPs of the CFH gene are translated into proteins. Two SNPs of the *CFH* gene (*rs*1061170 and *rs*800292) were retrieved from NCBI dsSNP (http;// www.ncbi.nlm.nih.gov/snp) and the amino acid sequence of *CFH* was obtained from Uniport knowledgebase (Uniprot ID: P08603) (http;//www.uniprot.org/). *CFH* SNPs *rs*1061170 and *rs*800292 result in a change in codon assignment from tyrosine (Y) to histidine (H) at amino acid positions 402 and from isoleucine (I) to valine (V) at position 62. For the selection of target peptides in the MRM assay, tryptic peptides unique to each protein isoform were identified from the UniProt database using the BLAST search tool (https://www.uniprot.org/blast/) (Table 1). Stable isotope-labeled standard (SIS) peptides with the same amino acid sequence as the target peptides, but incorporating C-terminal [^13^C_6_, ^15^N_4_] arginine (> 95% purity) were purchased from JPT Technologies (Berlin, Germany). The SIS peptides are used as quantitative standards in a mass spectrometer as they are physicochemically identical to the target peptides and have only a difference in mass. For each peptide, amino acid analysis was performed, and the absolute amount of peptide was provided by the vendor. The SIS peptides were re-solubilized in 20% acetonitrile with 0.1% formic acid to prepare a 1 mg/mL stock solution. Two other dilution series (50 and 100 ng/mL) were prepared to optimize the MRM assay. All solutions were stored at − 80 °C until use.

**Table 1:** Selection and optimization of the tryptic peptide to infer single nucleotide polymorphism genotype in multiple reaction monitoring assays.

Variant		SNP	SAAV	Target peptide	Endogenous SIS	Q1 (m/z)	Q3 (m/z)	Q1 z	Q3 z	Ion	DP	CE
*rs*1061170	*CFH* Y402	… AAT CAA AAT TAT GGA AGA …	.NQNYGR	YFPYLENGYNQNYGR	Endogenous	1029.4	868.1	+ 2	+ 2	y14	75	45
					SIS	1034.4	872.9					
	*CFH* H402	… AAT CAA AAT CAT GGA AGA …	.NQNHGR	YFPYLENGYNQNHGR	Endogenous	678.0	781.5	+ 3	+ 2	y13	71	29
					SIS	681.3	786.4					
*rs*800292	*CFH* V62	… CTT GGA AAT ATA ATA ATG …	. LGNIIM	SLGNIIMVR	Endogenous	574.8	678.4	+ 2	+ 1	y5	76	27
					SIS	579.8	688.4					
	*CFH* I62	… CTT GGA AAT GTA ATA ATG …	. LGNVIM	SLVNVIMVR	Endogenous	581.8	678.4	+ 2	+ 1	y5	76	29
					SIS	586.8	688.4					

SNP, single nucleotide polymorphism; SAAV, single amino acid variant; SIS, stable isotope-labeled; m/z, mass to charge; DP, declustering potential; CE, collision energy; CFH, complement factor H; T, thymine; C, cytosine; A, adenine; G, guanine; Y, tyrosine; H, histidine; I, isoleucine; V, valine.

### Optimization of MRM assay

We experimentally optimized most of the empirical parameters of the MRM assay by directly infusing the SIS peptides into a 5500 Qtrap mass spectrometer equipped with a turbo v ion source (SCIEX, Foster City, CA). First, the intensities of the doubly and triply charged ions (initial Q1) were scanned, and the optimal declustering potential (DP) voltage was determined by ramping the DP, and the dominant precursor ions (Q1) were then measured with the optimal DP voltage (final Q1). Second, at the optimized Q1 and DP parameters, we initially measured 10 highly intense product ions of a precursor ion using the base collision energy (CE) and then ramped the CE voltages to find the best CE values. Subsequently, we obtained the optimal collision cell exit potential (CXP) values of the selected 10 product ions. All optimized data were collected and compared to the theoretical spectra, and three highly intense y-ions were used for subsequent MRM assays (Table 1 and Supplementary Fig. S1).

### Sample preparation for MRM assay

The plasma (2 µL) sample was buffered with 50 µL of 8 M urea in 50 mM ammonium bicarbonate (ABC). The sample was treated using 7 µL of 100 mM tris (2-carboxyethyl) phosphine and 7 µL of 200 mM chloroacetamide at 37 °C for 1 h in the dark. Urea was diluted to 1 M with 50 mM ABC before trypsin (Promega, Madison, WI, USA) digestion at 1:50 enzyme: substrate ratio and incubated at 37 °C for 16 h with mixing on a shaker at 600 rpm. Formic acid was added at a final concentration of 0.5% to stop the digestion reaction. The mixture was desalted using a Sep-Pak tC18 96-well plate (Waters, Milford, MA, USA) and dried using a vacuum centrifuge (miVac Duo Concentrator; Genevac, Suffolk, UK). Dried samples were stored in a deep freezer at − 80 °C until use.

### LC-MRM-MS analysis

Peptide separation was performed using an ACQUITY UPLC M-class (Waters). Mobile phase A was 0.1% formic acid in the water, and mobile phase B was 0.1% formic acid in acetonitrile. Samples were reconstituted with 40 µL of SIS peptide mixture in mobile phase A, injected with a full sample loop injection of 5 µL, and separated in an ACQUITY UPLC peptide CSH C18 column (1 mm i.d., 10 cm length, pore size 130 Å, particle size 1.7 µm; Waters). A gradient with a flow rate of 20 µL/min and 5% B for 3 min, 5–25% B for 22 min, 25–60% for 1 min, 60–60% for 1 min, 60–5% B for 1 min, and 5% B for 5 min, followed by column washing with 80% B for 5 min, 80% to 5% for 6 min, 5–80% for 6 min, and re-equilibration with 5% B for 13 min. MRM analysis was performed using a 5500 Qtrap mass spectrometer. The MS detection was carried out in positive mode with the following parameters: ion spray voltage of 5500 V, curtain gas at 20 psi, nebulizer gas at 25 psi, heating gas at 20 psi, resolution at 0.7 Da (unit) for Q1/Q3, interface temperature at 400 °C, and scan mass range 300–1250 m/z QQQ mode. Quantification experiments were performed using the scheduled MRM mode. The MRM detection window time was 120 s, and the cycle time was 1.5 s. The mass spectrometer was operated with Analyst software (Version 1.6.2, SCIEX), which generated MRM-MS data with the file name *.wiff.

### Quantitative MRM assay

The concentration of the endogenous target peptide can be calculated by comparing the peak areas of the peptide and the spiked SIS peptide resulting from the LC-MRM-MS analysis. Peak areas were integrated into the Analyst software by setting a three points Gaussian smooth width, 30 s RT window, 500 cps minimum peak height, and one point peak splitting factor. For an accurate quantification, analytical validation was performed using calibration curves, specificity, precision and accuracy^13^. The calibration curves were used to predict the concentration of targets and to determine the limit of quantification (LOQ), the lowest concentration within a coefficient of variation (CV) of ≤ 20% with a coefficient of determination (R^2^) of 0.99 (Supplementary Fig. S2). Specificity was calculated by comparing endogenous analytes and internal standards in double blank (DB), specificity analytes (S.A), blank and low QC samples. Under optimized MRM conditions, no interference was observed for each sample in the target peptide (Supplementary Table S1). To evaluate precision and accuracy, we prepared four types of quality control (QC) samples of different concentrations. For intra- and inter-day assays, each concentration level was tested for three consecutive days in five replicates. Both intra- and inter-day QC samples at each concentration for all four analytes were acceptable (Supplementary Table S2 and S3).

### Statistical analysis

The statistical differences in age, sex, smoking status, and the genotype of *CFH SNP rs*1061170 and *rs*800292 between the normal controls and AMD groups were evaluated using the Chi-square test and Mann–Whitney test. The correlation between the plasma concentration of tyrosine and histidine and the genotypes of *CFH* SNP *rs*1061170 was evaluated using Kruskal–Wallis test. The correlation between the plasma concentration of valine and isoleucine and the genotypes of *CFH* SNP *rs*800292 was also evaluated using Kruskal–Wallis test. Scatter plots were drawn to visualize the correlation between the plasma tyrosine/histidine (Y/H) ratio and the genotypes of *CFH* SNP *rs*1061170 and between plasma valine/isoleucine (V/I) ratio and the genotypes of *CFH* SNP *rs*800292. Cut-off values of plasma *CFH* variant ratios were set to best delineate the *CFH* genotypes, and the correlation between MRM-based and DNA-based genotypes of *CFH* was evaluated. Statistical significance was defined as a *P* value of < 0.05. All statistical analyses were performed using SPSS (version 24.0; SPSS Inc., Chicago, USA).

## Results

In our study, the number of cases was 26 in the control group and 120 in the AMD group. The average age of the subjects was 68.4 ± 9.0 years. Among the patients with AMD, intermediate AMD was observed in 55 patients (45.8%), and nAMD was observed in 65 patients (54.2%). There were no statistical differences between the control and AMD groups in age, sex, and smoking status (*P* = 0.092, 0.840, and 0.303, respectively).

The distribution of alleles in *CFH* SNP *rs*1061170 was 121 for the TT allele (82.9%), 24 for the TC allele (16.4%), and 1 for the CC allele (0.7%). The distribution of alleles in *CFH* SNP *rs*800292 was 72 for the GG allele (49.3%), 57 for the AG allele (39.0%), and 17 for the AA allele (11.6%) (Table 2).

**Table 2:** The baseline demographics and distribution of genotype (complement factor H single nucleotide polymorphism of *rs*1061170 and *rs*800292) in controls and age-related macular degeneration groups.

Variables	Total (N = 146)	Normal controls (N = 26)	AMD groups (N = 120)
Age, years, mean ± SD	68.4 ± 9.0	60.1 ± 6.8	70.2 ± 8.5
**Sex, n (%)**			
Male	76 (52.1)	14 (53.8)	62 (51.7)
Female	70 (47.9)	12 (46.2)	58 (48.3)
**Types of AMD, n (%)**			
Intermediate AMD	55 (45.8)	–	55 (45.8)
Neovascular AMD	65 (54.2)	–	65 (54.2)
***CFH rs*1061170, n (%)**			
TT	121 (82.9)	19 (73.1)	102 (85.0)
TC	24 (16.4)	7 (26.9)	17 (14.2)
CC	1 (0.7)	0 (0.0)	1 (0.8)
***CFH rs*800292, n (%)**			
GG	72 (49.3)	11 (42.3)	61 (50.8)
AG	57 (39.0)	11 (42.3)	46 (38.3)
AA	17 (11.6)	4 (15.4)	13 (10.8)

SD, standard deviation; AMD, age-related macular degeneration; CFH, complement factor H.

**Figure 1:**
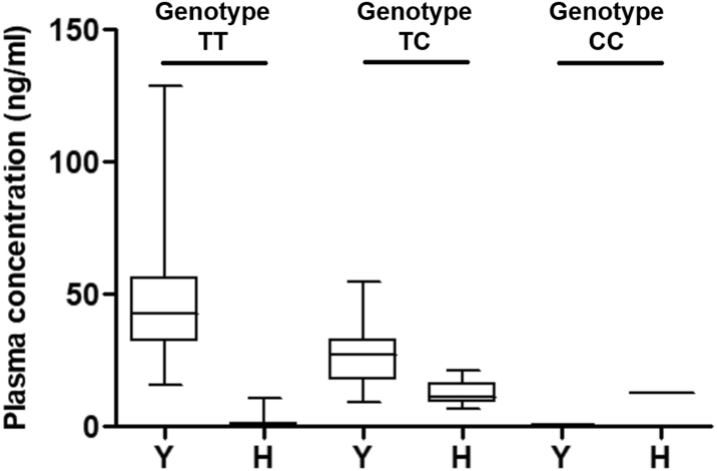
The plasma concentrations of complement factor H (CFH) variants with tyrosine and histidine by measuring multiple reaction monitoring assays according to *CFH* single nucleotide polymorphism *rs*1061170. Y, tyrosine; H, histidine.

**Figure 2:**
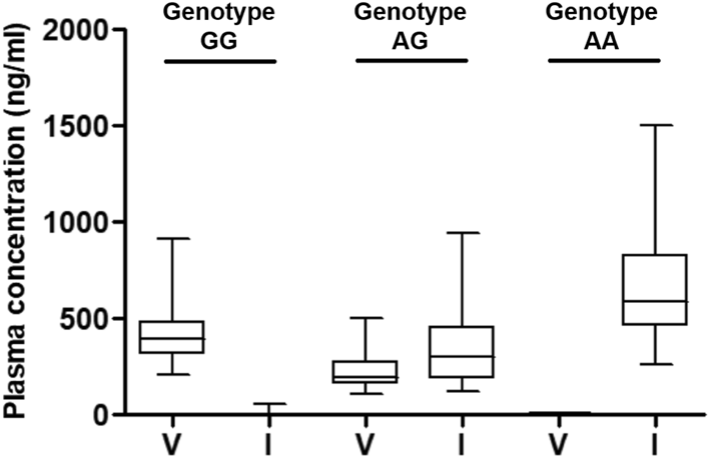
The plasma concentrations of complement factor H (CFH) variants with valine and isoleucine by measuring multiple reaction monitoring assays according to *CFH* single nucleotide polymorphism *rs*800292. V, valine; I, isoleucine.

The average plasma concentrations of tyrosine and histidine in the MRM assay according to *CFH* SNP *rs*1061170 genotypes were as follows: Tyrosine: 46.2 ± 18.4 ng/mL (TT allele), 27.0 ± 11.8 ng/mL (TC allele), 0.0001 ng/mL (CC allele); histidine, 0.861 ± 1.076 ng/mL (TT allele), 12.6 ± 4.5 (TC allele), and 12.7 ng/mL (CC allele) (Table 3, Fig. 1). There was a statistically significant difference between the plasma concentrations of CFH with tyrosine and histidine and the genotypes of *CFH* SNP *rs*1061170 (*P* < 0.001). The average plasma concentrations of valine and isoleucine in the MRM assay according to *CFH* SNP *rs*800292 were as follows: Valine: 423.1 ± 152.2 ng/mL (GG allele), 225.2 ± 93.8 (AG allele), 0.818 ± 1.017 (AA allele), isoleucine—3.1 ± 9.3 (GG allele), 341.3 ± 183.5 (AG allele), and 683.4 ± 354.5 (AA allele) (Table 4, Fig. 2). There was a statistically significant difference between the plasma concentrations of valine and isoleucine and the genotypes of *CFH* SNP *rs*800292 (*P* < 0.001).

**Table 3:** The plasma level of complement factor H variants by measuring multiple reaction monitoring assay and distribution, matching according to the genotype of complement factor H single nucleotide polymorphism *rs*1061170.

*rs*1061170	TT	TC	CC
Amino acid	Y	Y/H	H
**N**			
Total	121	24	1
Control	19	7	0
AMD	102	17	1
Plasma CFH (Y) concentration (ng/mL), mean ± SD	46.2 ± 18.4	27.0 ± 11.8	0.0001
Plasma CFH (H) concentration (ng/mL), mean ± SD	0.861 ± 1.076	12.6 ± 4.5	12.7
Y/H ratio, mean ± SD	91.301 ± 68.915	2.218 ± 0.882	0.00000785453

*CFH* (Y)/*CFH* (H) ratio	Total (control/AMD)	Total (control/AMD)	Total (control/AMD)
< 1.00	0 (0/0)	0 (0/0)	1 (0/1)
1.00–4.428	0 (0/0)	24 (7/17)	0 (0/0)
> 4.428	121 (19/102)	0 (0/0)	0 (0/0)
Gene/MRM matching	100%	100%	100%

Y, tyrosine; H, histidine; AMD, age-related macular degeneration; MRM, multiple reaction monitoring; SD, standard deviation.

**Table 4:** The plasma level of complement factor H variants by measuring multiple reaction monitoring assay and distribution, matching according to the genotype of complement factor H single nucleotide polymorphism *rs*800292.

*rs*800292	GG	AG	AA
Amino acid	V	V/I	I
**N**			
Total	72	57	17
Control	11	11	4
AMD	61	46	13
Plasma V concentration (ng/mL), mean ± SD	423.1 ± 152.2	225.2 ± 93.8	0.818 ± 1.017
Plasma I concentration (ng/mL), mean ± SD	3.1 ± 9.3	341.3 ± 183.5	683.4 ± 354.5
V/I ratio, mean ± SD	606.906 ± 641.983	0.711 ± 0.138	0.001 ± 0.002

*CFH* (V)/*CFH* (I) ratio	Total (control/AMD)	Total (control/AMD)	Total (control/AMD)
< 0.0089	0 (0/0)	0 (0/0)	17 (4/13)
0.0089–1.08	0 (0/0)	57 (11/46)	0 (0/0)
> 1.09	72 (11/61)	0 (0/0)	0 (0/0)
Gene/MRM matching	100%	100%	100%

V, valine; I, isoleucine; AMD, age-related macular degeneration; MRM, multiple reaction monitoring; SD, standard deviation.

**Figure 3:**
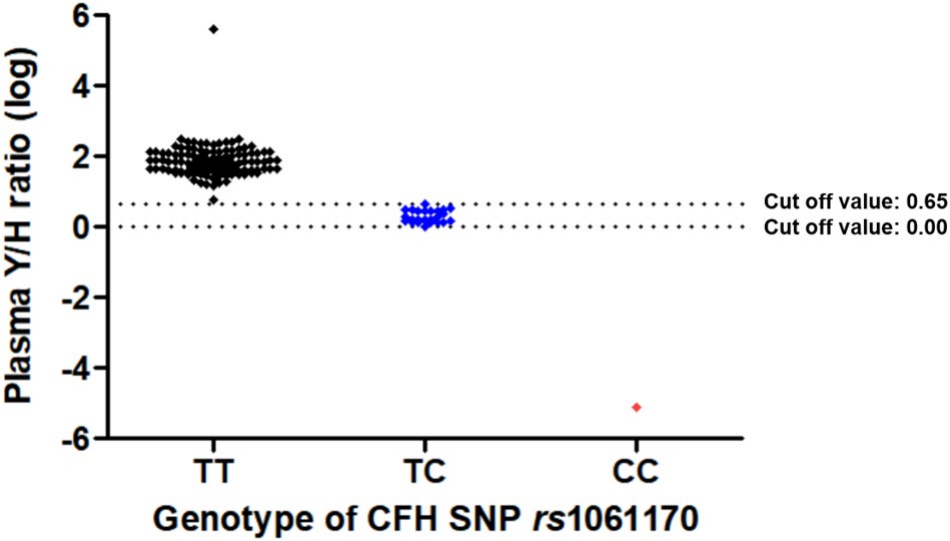
The distribution of logarithm plasma CFH tyrosine/histidine variant protein ratio according to the genotype of complement factor H single nucleotide polymorphism *rs*1061170. CFH, complement factor H; SNP, single nucleotide polymorphism; Y, tyrosine; H, histidine; log, logarithm.

**Figure 4:**
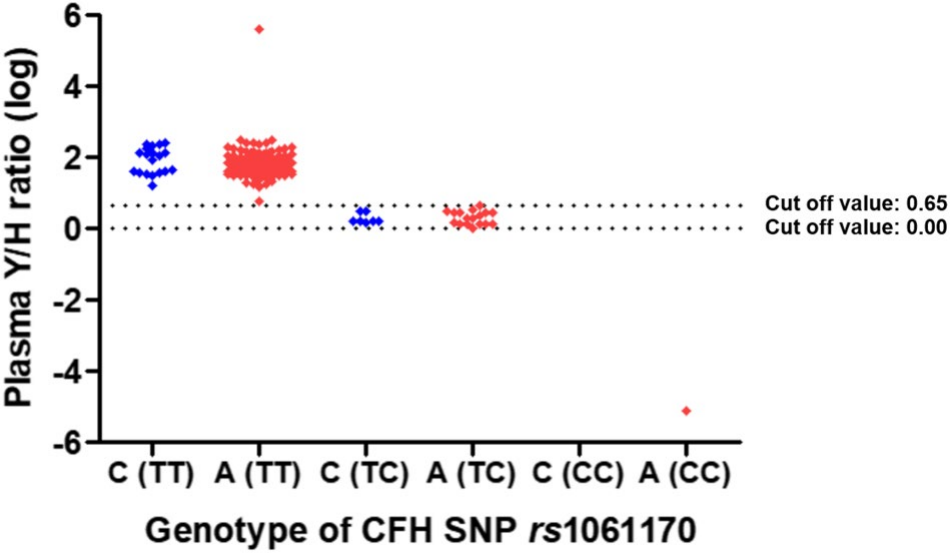
The distribution of logarithm plasma CFH tyrosine/histidine variant protein ratio according to the genotype of complement factor H single nucleotide polymorphism *rs*1061170 and the presence of age-related macular degeneration. C, control; A, age-related macular degeneration; CFH, complement factor H; SNP, single nucleotide polymorphism; Y, tyrosine; H, histidine; log, logarithm.

The correlation between the plasma CFH tyrosine/histidine (Y/H) variant protein ratio and the genotypes of *CFH* SNP *rs*1061170 are depicted in Fig. 3. The average plasma CFH Y/H ratio according to the genotypes of *CFH* SNP *rs*1061170 was as follows: TT, 91.301 ± 68.915 (5.804–403,100.775); TC, 2.218 ± 0.882 (1.059–4.428); CC: 0.00000785453. We set the cut-off values as 4.428 and 1.00, to match the plasma Y/H ratio and the SNP genotypes. (TT: Y/H ratio > 4.428; TC: 1.00 ≤ Y/H ratio ≤ 4.428; CC: Y/H ratio < 1.00) The distribution of plasma CFH Y/H ratio by measuring MRM assay and genotype of *CFH* SNP *rs*1061170 was exactly matched (100%) (Table 3, Fig. 4).

**Figure 5:**
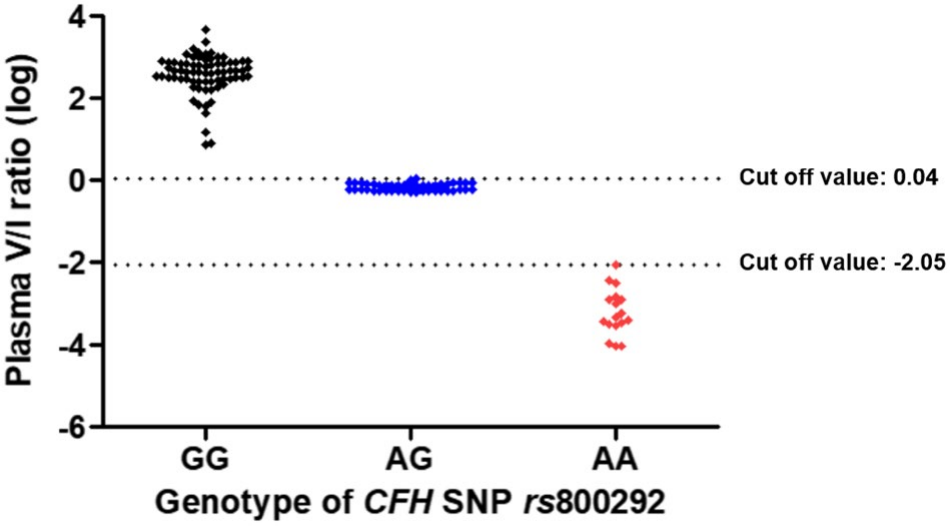
The distribution of logarithm plasma CFH valine/isoleucine varinat protein ratio according to the genotype of complement factor H single nucleotide polymorphism *rs*800292. CFH, complement factor H; SNP, single nucleotide polymorphism; V, valine; I, isoleucine; log, logarithm.

**Figure 6:**
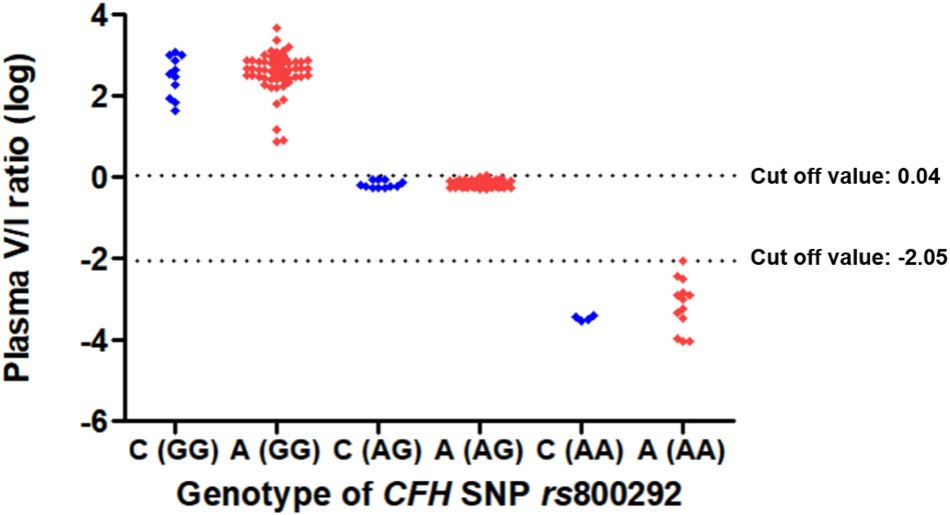
The distribution of logarithm plasma CFH valine/isoleucine variant protein ratio according to the genotype of complement factor H single nucleotide polymorphism *rs*800292 and the presence of age-related macular degeneration. C, control; A, age-related macular degeneration; CFH, complement factor H; SNP, single nucleotide polymorphism; V, valine; I, isoleucine; log, logarithm.

The correlation between the plasma CFH valine/isoleucine (V/I) variant protein ratio and the genotypes of *CFH* SNP *rs*800292 is depicted in Fig. 5. The average plasma CFH V/I ratio according to the genotypes of *CFH* SNP *rs*800292 was as follows: GG, 606.906 ± 641.983 (7.854–4654.144); GA, 0.711 ± 0.138 (0.519–1.090); AA, 0.001 ± 0.002 (0.0001–0.009). We set the cut-off values as 1.09 and 0.0089 to match the plasma V/I ratio and SNP genotypes (GG: V/I ratio > 1.09; AG: 0.0089 ≤ V/I ratio ≤ 1.08; AA: V/I ratio < 0.0089). The distribution of plasma V/I ratio by measuring MRM assay and genotype of *CFH* SNP *rs*800292 was exactly matched (100%) (Table 4, Fig. 6). The plasma concentrations of *CFH* protein variants could accurately predict the genotypes of *CFH* SNPs rs1061170 and rs800292.

## Discussion

In our study, we measured the plasma concentrations of CFH variants using MRM analysis and correlated the CFH variant ratios with the genotypes of *CFH* SNPs *rs*1061170 and *rs*800292. We identified an exact match between the genotypes of *CFH* SNPs *rs*1061170 and *rs*800292 and the ratios of plasma levels of CFH variants. Thus, the quantification of plasma CFH variants using the MRM assay may be a useful alternative to DNA-based genotyping of the two *CFH* SNPs.

The SNPs in *the CFH* and *age-related maculopathy susceptibility 2 (ARMS2)* genes have shown the greatest impact on risk assessment and the pathogenic mechanism of AMD^6,14–16^. CFH is present at relatively high concentrations in the blood. CFH is produced primarily in the liver and circulates throughout the body including the retina through the blood^17^. The abundant concentration of CFH in the blood enabled the measurement of CFH variants and the correlation with the corresponding genotypes. However, in the case of *ARMS2*, no protein has been found in the blood; thus, proteomic analysis is not feasible. Among other genes related to AMD, genes that produce proteins present in the blood can be analyzed using the MRM assay in the same way. However, the major risk SNPs of AMD are *CFH* and *ARMS2* (odds ratio: *ARMS2* SNP *rs*10490924, 3.13; *CFH* SNP *rs*1061170, 2.74; *CFH* SNP *rs*800292, 2.43), while the other known SNPs related to AMD have insufficient clinical significance (e.g. odds ratio: 2.82 for *CFB rs*4151667; 2.31 for *CFB rs*438999; 1.58 for *SYN3/TIMP3* rs9621532; 1.52 for *C3 rs*2230199)^18^. In the previous studies, in Caucasians, Y402H (*CFH* SNP *rs*1061170) was reported to be highly related to AMD, while in Asians, I62V (*CFH* SNP *rs*800292) was reported to be highly related to AMD^9,19–25^. Thus, proteomic identification of *CFH* SNPs Y402H and I62V can be applied with value to both ethnicities, Asians and Caucasians.

A recent study by Zhang et al. quantified plasma *CFH* variants (Y402H, I62V) and CFH-related proteins (complement factor-related protein 1 to 5) using the multiplexed targeted mass spectrometry assay^11^. They analyzed the blood samples of participants in the Age, Gene/Environment Susceptibility-Reykjavik study and showed plasma CFH variant concentrations by inferred genotypes. Thus, they did not correlate plasma CFH protein concentrations with the true genotypes of the *CFH* gene SNPs. Additionally, the study was a population-based study that did not define patients with AMD and controls; thus, the actual clinical application of the MRM assay was not tested before.

In our study, we measured the plasma levels of CFH variants using MRM analysis and analyzed the genotypes of *CFH* SNPs *rs*1061170 and *rs*800292. Importantly, we proposed cut-off values for protein variant ratios for the discrimination of SNPs and identified an exact match between DNA-based genotypes and MRM-based genotypes of *CFH* SNPs *rs*1061170 and *rs*800292. The further advantages of proteomic genotyping can be suggested as follows: (1) It can be combined with other protein markers up to approximately 100 in a single MRM assay. Thus, additional proteomic analysis of plasma proteins can be performed simultaneously with minimal additional costs; (2) In the future, the risk SNPs of various genes that encode proteins expressed in the blood can be added for AMD and other diseases. This will shift the paradigm of genotyping from DNA-based genetic assays to protein-based multi-omics.

Our study has several limitations. First, our study was a retrospective, observational, single-center design. Second, the relatively small sample size of one ethnic group may be another limitation. Future validation including larger samples of different ethnicities is needed. However, our study showed an exact match between the proteomic genotypes defined by the concentration of plasma CFH variants and the DNA-based genotypes of *CFH* SNPs. To the best of our knowledge, this is the first study to suggest a clinically applicable method to identify the genotypes of CFH using proteomic MRM assay in a cohort of patients with AMD and controls.

In conclusion, the measurement of plasma CFH protein variants using the MRM assay can accurately predict the genotypes of *CFH* SNPs of Y402H and I62V. Proteomic genotyping of CFH using the MRM assays can be used without DNA-based genetic tests for the risk assessment of AMD.

## Supplementary Information

Supplementary Information 1.

Supplementary Information 2.

## Data Availability

The datasets generated during and/or analysed during the current study are available in the Harvard Dataverse repository, [https://dataverse.harvard.edu/api/access/datafile/6413273].
