# Supplementary material for: Proteomic genotyping of SNP of Complement Factor H (CFH) Y402H and I62V using multiple reaction monitoring (MRM) assays
Source: Sci Rep. 2022 Nov 15;12:19587. doi: 10.1038/s41598-022-20936-8 (PMC9666549; doi:10.1038/s41598-022-20936-8)
Supplement: Supplementary file 1 — Supplementary Information 1. [file 41598_2022_20936_MOESM1_ESM.docx]

| CFH Variant | | Analyte / SIS | Peak area | | | | | | | | | | | | | | |
| --- | --- | --- | --- | --- | --- | --- | --- | --- | --- | --- | --- | --- | --- | --- | --- | --- | --- |
|  |  |  | **DB _01** | **DB_02** | **DB_03** | **DB_04** | **DB_05** | **DB_06** | **S.A _01** | **S.A_02** | **S.A_03** | **Blank _01** | **Blank_02** | **Blank_03** | **Low**  **QC_01** | **Low**  **QC_02** | **Low**  **QC_03** |
| *rs*1061170 | Y402 | Analyte | 0 | 0 | 0 | 0 | 0 | 0 | 26000 | 30000 | 29600 | 0 | 0 | 0 | 30800 | 31500 | 31600 |
|  |  | SIS | 0 | 0 | 0 | 0 | 0 | 0 | 0 | 0 | 0 | 66100 | 61800 | 59500 | 4620 | 4880 | 5040 |
|  | H402 | Analyte | 0 | 0 | 0 | 0 | 0 | 0 | 22900 | 22300 | 21000 | 0 | 0 | 0 | 24100 | 28100 | 23700 |
|  |  | SIS | 0 | 0 | 0 | 0 | 0 | 0 | 0 | 0 | 0 | 686000 | 642000 | 629000 | 481000 | 470000 | 493000 |
| *rs*800292 | V62 | Analyte | 0 | 0 | 0 | 0 | 0 | 0 | 1680000 | 1830000 | 1800000 | 0 | 0 | 0 | 1770000 | 1740000 | 1780000 |
|  |  | SIS | 0 | 0 | 0 | 0 | 0 | 0 | 0 | 0 | 0 | 6540000 | 6210000 | 6130000 | 3810000 | 3630000 | 3810000 |
|  | I62 | Analyte | 0 | 0 | 0 | 0 | 0 | 0 | 978000 | 1150000 | 1120000 | 0 | 0 | 0 | 1120000 | 1120000 | 1120000 |
|  |  | SIS | 0 | 0 | 0 | 0 | 0 | 0 | 0 | 0 | 0 | 4950000 | 4690000 | 4890000 | 3450000 | 3400000 | 3450000 |
| Abbreviation: CFH, complement factor H; SIS, stable isotope-labeled standard; DB, double blank; S.A, specific analytes, QC, quality control; Y, tyrosine; H, histidine; V, valine; I, isoleucine. | | | | | | | | | | | | | | | | | |

**Supplementary Table S1.** Analytical specificity of complement factor H variants peptide.

| CFH Variant | | Level | Measurement value (ng/mL) | | | | | Mean (ng/mL) | SD | Accuracy (%) | Precision (%) |
| --- | --- | --- | --- | --- | --- | --- | --- | --- | --- | --- | --- |
|  |  |  | **1** | **2** | **3** | **4** | **5** |  |  |  |  |
| *rs*1061170 | Y402 | QC1 | 1495.00 | 1595.00 | 1325.00 | 1605.00 | 1565.00 | 1517.00 | 115.63 | 96.93 | 7.62 |
|  |  | QC2 | 677.00 | 673.00 | 674.00 | 678.00 | 676.00 | 675.60 | 2.07 | 99.94 | 0.31 |
|  |  | QC3 | 151.40 | 155.00 | 155.60 | 155.60 | 157.80 | 155.08 | 2.32 | 99.67 | 1.49 |
|  |  | QC4 | 59.50 | 59.50 | 59.00 | 57.00 | 59.50 | 58.90 | 1.08 | 98.99 | 1.84 |
|  | H402 | QC1 | 14.35 | 14.40 | 14.55 | 14.05 | 14.50 | 14.37 | 0.20 | 99.79 | 1.36 |
|  |  | QC2 | 4.60 | 4.55 | 4.98 | 4.93 | 4.92 | 4.80 | 0.20 | 97.48 | 4.25 |
|  |  | QC3 | 3.56 | 3.70 | 3.56 | 3.60 | 3.48 | 3.58 | 0.08 | 100.56 | 2.23 |
|  |  | QC4 | 3.78 | 3.73 | 3.74 | 3.41 | 3.71 | 3.67 | 0.15 | 98.55 | 4.04 |
| *rs*800292 | V62 | QC1 | 56.00 | 55.00 | 53.50 | 52.50 | 54.00 | 54.20 | 1.35 | 100.37 | 2.49 |
|  |  | QC2 | 55.30 | 52.00 | 50.70 | 51.60 | 52.30 | 52.38 | 1.74 | 100.73 | 3.32 |
|  |  | QC3 | 53.60 | 51.40 | 50.00 | 50.00 | 49.00 | 50.80 | 1.78 | 101.60 | 3.51 |
|  |  | QC4 | 56.50 | 53.00 | 53.50 | 54.00 | 52.50 | 53.90 | 1.56 | 100.75 | 2.89 |
|  | I62 | QC1 | 39.35 | 38.05 | 40.40 | 40.35 | 38.30 | 39.29 | 1.10 | 99.85 | 2.81 |
|  |  | QC2 | 32.90 | 34.40 | 34.50 | 33.90 | 33.90 | 33.92 | 0.63 | 100.06 | 1.87 |
|  |  | QC3 | 32.60 | 34.00 | 32.00 | 32.00 | 33.00 | 32.72 | 0.83 | 100.37 | 2.54 |
|  |  | QC4 | 36.20 | 35.50 | 35.35 | 34.00 | 34.70 | 35.15 | 0.84 | 99.43 | 2.38 |
| Abbreviation: CFH, complement factor H; SD, standard deviation; QC, quality control; Y, tyrosine; H, histidine; V,valine; I, isoleucine. | | | | | | | | | | | |

**Supplementary Table S2.** Intra-day precision and accuracy of assays.

**Supplementary Table S3.** Inter-day precision and accuracy of assays.

| CFH Variant | | Level | Measurement value (ng/mL) | | | Mean (ng/mL) | SD | Accuracy (%) | Precision (%) |
| --- | --- | --- | --- | --- | --- | --- | --- | --- | --- |
|  |  |  | **Day 1** | **Day 2** | **Day 3** |  |  |  |  |
| *rs*1061170 | Y402 | QC1 | 1517.00 | 1565.00 | 1555.00 | 1545.67 | 25.32 | 99.40 | 1.64 |
|  |  | QC2 | 675.60 | 677.80 | 701.80 | 685.07 | 14.53 | 101.07 | 2.12 |
|  |  | QC3 | 155.08 | 167.32 | 169.88 | 164.09 | 7.91 | 98.07 | 4.82 |
|  |  | QC4 | 58.90 | 57.70 | 58.50 | 58.37 | 0.61 | 99.77 | 1.05 |
|  | H402 | QC1 | 14.37 | 14.53 | 14.56 | 14.49 | 0.10 | 99.70 | 0.71 |
|  |  | QC2 | 4.80 | 4.78 | 4.83 | 4.80 | 0.02 | 100.13 | 0.45 |
|  |  | QC3 | 3.58 | 3.95 | 3.99 | 3.84 | 0.22 | 97.23 | 5.86 |
|  |  | QC4 | 3.67 | 3.71 | 3.76 | 3.71 | 0.04 | 100.04 | 1.17 |
| *rs*800292 | V62 | QC1 | 54.20 | 55.10 | 54.00 | 54.43 | 0.59 | 100.43 | 1.08 |
|  |  | QC2 | 52.38 | 51.64 | 51.44 | 51.82 | 0.50 | 100.35 | 0.96 |
|  |  | QC3 | 50.80 | 51.72 | 51.16 | 51.23 | 0.46 | 100.13 | 0.91 |
|  |  | QC4 | 53.90 | 53.10 | 53.50 | 53.50 | 0.40 | 100.00 | 0.75 |
|  | I62 | QC1 | 39.29 | 39.60 | 39.64 | 39.51 | 0.19 | 99.77 | 0.48 |
|  |  | QC2 | 33.92 | 33.46 | 33.50 | 33.63 | 0.25 | 100.38 | 0.76 |
|  |  | QC3 | 32.72 | 33.36 | 33.84 | 33.31 | 0.56 | 99.84 | 1.69 |
|  |  | QC4 | 35.15 | 35.88 | 35.70 | 35.58 | 0.38 | 99.65 | 1.07 |
| Abbreviation: CFH, complement factor H; SD, standard deviation; QC, quality control; Y, tyrosine; H, histidine; V, valine; I, isoleucine. | | | | | | | | | |

**
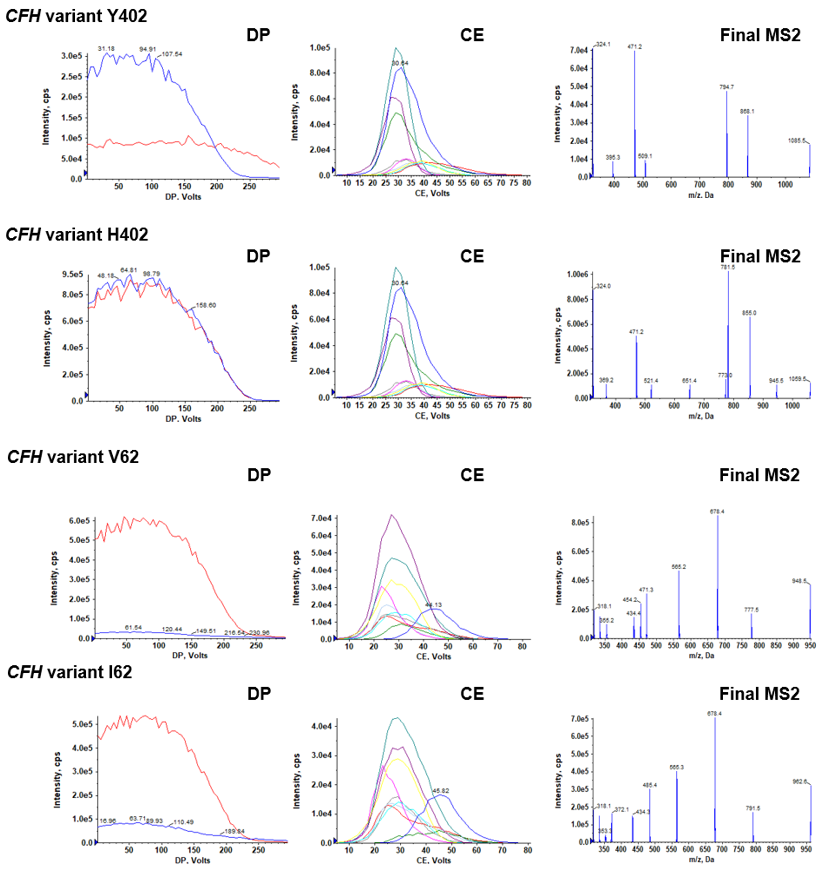
**

**Supplementary Figure S1. Optimization of target peptide of complement factor H variants.** The optimized declustering potential (DP) for doubly (blue) and triply (red) charged ion is found by ramping the voltage. The initial product ion scan for the dominant charge stage is performed with the optimized DP and the 10 most intense production are selected for collision energy optimization. Collisional cell exit potential is also optimized for the 10 most intense transitions. As a final step, the product ions are scanned using all of the optimized instrumental parameters. CFH, complement factor H; Y, tyrosine; DP, declustering potential; CE, collision energy; H, histidine; V, valine; I, isoleucine.


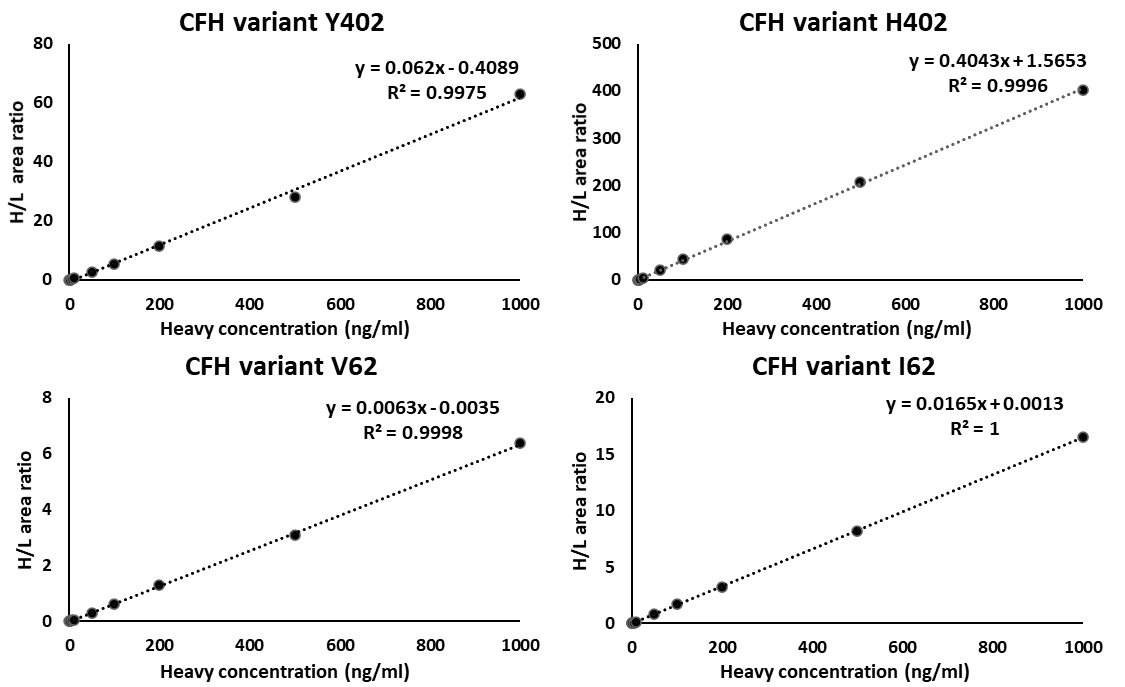


**Supplementary Figure S2.** **Calibration curve of complement factor H variant peptides.** Stable isotope-labeled standard (SIS) peptides with complement factor H (CFH) Y402 (y14++), H402 (y13++), V62 (y5+), and I62 (y5+) were quantified in the range of 0.1- 1000 ng/ml and the quantified multiple reaction monitoring (MRM) signal was plotted against the amount of the SIS peptide. A dilution series of the SIS peptides was analyzed in triplicated MRM run and the resultant MRM peak areas were plotted as a function of the peptide amount. The straight line within the plots represents linear response range with coefficient determination (R^2^) ≥ 0.99 and coefficient variation (CV) ≤ 20%, respectively. The analyzed data are included in Supplementary Data. CFH, complement factor H; Y, tyrosine; H, histidine; V, valine; I, isoleucine.
